# Medical Items

**Published:** 1892-10

**Authors:** 


### *MEDICAL ITEMS.*

UP to the first of September there are said to have been 150,000 deaths from cholera in Russia alone.

---

DR. C. D. HURT, of Columbus, has taken up his residence in Atlanta. Office in the Equitable Building.

---

WE hear that the S. S. S. man of Atlanta has gone on a visit to Hot Springs, Arkansas, for his "rheumatism."

---

DR. P. R. CORTELYOW, of Marietta, passed through the city some days ago, *en route* for New York, where he will spend several weeks in hospital and clinical work.

---

DR. JOHN J. REESE, of Philadelphia, the eminent toxicologist and author of a well known work on Medical Jurisprudence, died September 3d, aged seventy-four.

---

THE *Charlotte Medical Journal* is the latest to enter the field of Journalism. It is published at Charlotte, N. C., by Drs. E. C. Register and J. C. Montgomery, and the numbers which we have seen are very creditable.

---

BY order of the council, the annual meeting of the Southern Surgical and Gynecological Association has been postponed from the 8th, 9th and 10th until the 15th, 16th and 17th of November. It was thought wise to change the time of the meeting from the fact that the 8th of November is the date of the presidential election. Everything points to a very successful session.

THE twentieth annual meeting of the American Public Health Association will be held in the City of Mexico, November 29th, 30th, and December 1st and 2d. This Association, which originally was confined to the United States, now embraces Canada and Mexico also.

---

DR. F. W. McRAE has been elected to the chair of Physiology in the Southern Medical College, this city, *vice* Dr. J. C. Olmsted, resigned. In the same college Drs. L. M. Crichton and C. D. Roy succeeded to the chairs of Nose and Throat, and Eye and Ear, in the place of Dr. A. G. Hobbs.

---

DR. THOS. F. WOOD, of Wilmington, N. C., associate editor of the *N. C. Medical Journal*, and secretary of the State Board of Health, died August 22d. Dr. Wood was one of the most prominent of North Carolina physicians, and had made many contributions to medical and sanitary science. His death was due to heart disease and aortic aneurism.

---

IN the *Cronica Medico-chirurgica de la Habana* Drs. Acosta and Rossi give the results of their bacteriological examination of certain Spanish bank notes. Their analysis showed the presence of large numbers of microbes on notes that had been for a long time in use, and on two notes they calculated that there were upwards of nineteen thousand. One species proved rapidly fatal to animals inoculated with it. When the ever-present and invisible army of microbes attack the public purse they begin to be more interesting than ever before, and the outcome will be watched with much solicitude.

We hope this item will stimulate our delinquent subscribers to surrender some of their infected and disease-bearing notes. All such currency can be sterilized in this office. We assume all risks.

NOT long since the wife of a prominent city official in New York, who gets a salary of ten thousand dollars a year, appeared at one of the dispensaries, where only the very poor are treated, with her child, for whom she sought advice and treatment. The case was of such a serious character that the dispensary surgeon advised hospital care, and the mother became frightened and said that she would pay for medical attention at her home.

---

A correspondent of the New York *Herald*, named Stanhope, recently submitted himself to inoculation with the virus of cholera at the Pasteur Institute in Paris. Now as a further test of the virtue of the inoculation he has entered the cholera hospital at Hamburg, where he expects to mingle intimately with cholera patients and run every risk of contracting the disease. This bold experiment, which has some suggestion of newspaper enterprise, may mean a great deal for humanity. Whatever the motive and whatever the result, Mr. Stanhope's courage will entitle him to the admiration of the world.

---

LIKE the rich man in Hades who wished to send back to Earth a word of warning to his brothers, the Iodide of Potash Club, of Hot Springs, Arkansas, has issued the following letter to those contemplating a visit to that specific health resort:

"Don't listen to any one who volunteers advice about doctors. No regular physician will require more than $5 in advance. If you have letters to a physician, deliver them in person. If drummers find you have such letters they will tell you the doctor is out of the city, dead, quit practicing, drunk, or something of the kind. Drummers on the trains, on the streets, or at the hotels or boarding houses will pretend that they are visitors."

